# Phylogenomics reveals deep relationships and diversification within phylactolaemate bryozoans

**DOI:** 10.1098/rspb.2022.1504

**Published:** 2022-11-09

**Authors:** Ahmed J. Saadi, Julian Bibermair, Kevin M. Kocot, Nickellaus G. Roberts, Masato Hirose, Andrew Calcino, Christian Baranyi, Ratcha Chaichana, Timothy S. Wood, Thomas Schwaha

**Affiliations:** ^1^ Department of Evolutionary Biology, Unit for Integrative Zoology, University of Vienna, Djerassiplatz 1, A-1030 Vienna, Austria; ^2^ Department of Biological Sciences and Alabama Museum of Natural History, University of Alabama, Tuscaloosa, AL 35487, USA; ^3^ School of Marine Biosciences, Kitasato University, Kitasato 1-15-1, Sagamihara-Minami, Kanagawa 252-0373, Japan; ^4^ Department of Environmental Technology and Management, Faculty of Environment, Kasetsart University, Bangkok, Thailand; ^5^ Department of Biological Sciences, Wright State University, Dayton, OH 45435, USA

**Keywords:** Bryozoa, Phylactolaemata, phylogeny, evolution, divergence times, freshwater bryozoans

## Abstract

Bryozoans are mostly sessile colonial invertebrates that inhabit all kinds of aquatic ecosystems. Extant bryozoan species fall into two clades with one of them, Phylactolaemata, being the only exclusively freshwater clade. Phylogenetic relationships within the class Phylactolaemata have long been controversial owing to their limited distinguishable characteristics that reflect evolutionary relationships. Here, we present the first phylogenomic analysis of Phylactolaemata using transcriptomic data combined with dense taxon sampling of six families to better resolve the interrelationships and to estimate divergence time. Using maximum-likelihood and Bayesian inference approaches, we recovered a robust phylogeny for Phylactolaemata in which the interfamilial relationships are fully resolved. We show Stephanellidae is the sister taxon of all other phylactolaemates and confirm that Lophopodidae represents the second offshoot within the phylactolaemate tree. *Plumatella fruticosa* clearly falls outside Plumatellidae as previous investigations have suggested, and instead clusters with Pectinatellidae and Cristatellidae as the sister taxon of Fredericellidae. Our results demonstrate that cryptic speciation is very likely in *F. sultana* and in two species of *Plumatella* (*P. repens* and *P. casmiana*). Divergence time estimates show that Phylactolaemata appeared at the end of the Ediacaran and started to diverge in the Silurian, although confidence intervals were large for most nodes. The radiation of most extant phylactolaemate families occurred mainly in the Palaeogene and Neogene highlighting post-extinction diversification.

## 1. Introduction

Bryozoans are suspension feeding, almost entirely colonial invertebrates that inhabit all kinds of aquatic environments with approximately 6000 extant and 15 000 fossil species [1,2]. All colonies consist of multiple individuals or iterated modules termed zooids, which each—in its simplest form—consists of a feeding tentacle crown or lophophore and u-shaped digestive tract that are retractable into the protective body wall [3]. Bryozoans are known from the early Cambrian of Australia, South China and western United States [4,5] and represent one of the three dominant groups of Palaeozoic fossils [6]. The phylum Bryozoa was divided by Woollacott and Zimmer [2] into three main taxa: Phylactolaemata, Stenolaemata and Gymnolaemata. The monophyly of these three classes has been supported by the molecular studies of Fuchs *et al*. [7], Waeschenbach *et al*. [8] and Orr *et al*. [9]. Phylactolaemata is recognized to be the earliest extant branch and as the sister clade of the remaining bryozoan clades [7–10]. Stenolaemata and Gymnolaemata were recently united by Schwaha *et al*. [11] in one clade called Myolaemata based on existing molecular studies and soft tissue morphology. Phylactolaemates are uncalcified and exclusively freshwater species while myolaemates are mainly calcified with a predominantly marine distribution [3]. Approximately 80 recent phylactolaemate species are described, having broad distributions with some cosmopolitan species [12]. They represent the largest forms of bryozoans both in terms of their individual zooids and also their colonies (e.g. *Pectinatella magnifica* colonies often grow to greater than 2 m in diameter) [3,13]. Phylactolaemates are morphologically distinct from other bryozoans by the horseshoe-shaped lophophore and by statoblasts which are encapsulated, asexually produced dormant stages [14–16]. Three main forms of statoblasts (floatoblasts, sessoblasts and piptoblasts) are recognized and their morphology is widely used for species identification [15]. Phylactolaemates are represented in the fossil record only through the rare preservation of statoblasts with the earliest known from the Upper Permian of Russia [17] and Late Triassic of South Africa [18].

Phylactolaemata was traditionally classified into six families: Cristatellidae, Fredericellidae, Lophopodidae, Pectinatellidae, Plumatellidae and Stephanellidae. A seventh family, Tapajosellidae was recently added by Wood & Okamura [19] but it is only defined based on statoblasts and no colonies have been found. Of these families, Cristatellidae, Pectinatellidae and Stephanellidae are monotypic families [3]. Cristatellidae and Pectinatellidae produce only a spiny type of floatoblast called spinoblasts while Stephanellidae produces both floatoblasts and sessoblasts [15]. Fredericellidae is the second largest phylactolaemate family, comprising approximately eight species belonging to two genera. Species of *Fredericella* produce only sessile, bean-shaped piptoblasts while *Internectella* produces both floatoblasts and sessoblasts [3]. Lophopodidae includes three genera and seven species, all of which produce floatoblasts only [3,12]. Finally, Plumatellidae, the most diverse family within the phylactolaemates, encompasses possibly seven genera with about 65 recent species [16] with almost all of them producing floatoblasts and sessoblasts [3].

Phylogenetic relationships within Phylactolaemata have long been controversial owing to their limited distinguishable features that reflect evolutionary relationships. Characters related to the pattern of colony growth and statoblasts morphology have been used as the basis for phylogenetic hypotheses within this class. In the early morphological studies of the phylactolaemates [14,20,21], species with rather simple and distinct branching colonies mainly represented by Fredericellidae and Plumatellidae were regarded as plesiomorphic groups, while those with compact, gelatinous colonies (Lophopodidae, Pectinatellidae and Cristatellidae) were regarded as derived groups. By contrast, molecular phylogenetic studies of Phylactolaemata based on a single gene or a combination of few loci [8,22,23] have reversed the morphology-based phylogenies by recovering the compact gelatinous forms as early branching lineages within Phylactolaemata. Although previous molecular phylogenies of phylactolaemate bryozoans [8,22–26] have improved our understanding of evolutionary relationships within Phylactolaemata, the interfamilial relationships within this class remain largely unresolved. Moreover, none of these studies have estimated the timing of the last common ancestor of extant phylactolaemate and the divergence of major clades.

Phylogenomic analyses, which leverage dozens to hundreds of molecular markers derived from genomes or transcriptomes, have become a powerful tool for inferring metazoan phylogenetic relationships. However, no phylogenomic study to date has investigated the evolutionary relationships within Phylactolaemata. Here, we employed a phylogenomic approach to reconstruct a broad-scale phylogenetic framework for the phylactolaemate bryozoans using transcriptomic data. We generated new transcriptome data for 31 bryozoans and combined this dataset with publicly available transcriptomes to investigate the interfamilial relationships of phylactolaemates. With the inclusion of seven fossil calibration points, we also present the first divergence time estimates incorporating a fully resolved topology for the entire bryozoan clade.

## 2. Material and methods

### (a) Taxon sampling, RNA extraction, library construction and sequencing

Our taxon sampling included 17 phylactolaemate species representing all phylactolaemate families except Tapajosellidae. A range of non-phylactolaemate bryozoans: three cyclostomes, six ctenostomes and five cheilostomes were also included along with two phoronids and two brachiopods as outgroups. Thirty-two transcriptomes, including 31 bryozoans and one phoronid, were newly generated while 12 transcriptomes were sampled from publicly available data as either raw sequence reads (*Fredericella sultana* 5, 6 & 7 [27]; *Flustrellidra corniculata* and *Heteropora pacifica* [28]; *Laqueus californicus* and *Membranipora membranacea* [29]; *Phoronis vancouverensis* [30]; *Schizoporella errata* and *Watersipora subtorquata* [31]) or assembled transcriptomes (*Bugulina stolonifera* [32] and *Lingula anatina* [33]); details of the specimens, sources of publicly available sequences and GenBank Bioproject accession number are given in electronic supplementary material, table S1. Digital vouchers for most species of plumatellids and fredericellids are available on Dryad digital repository.

For most of the newly sequenced taxa, RNA was extracted and purified from RNAlater-preserved samples at The University of Vienna using the RNeasy Plus Mini kit (both Qiagen, Hilden, Germany) following the manufacturer's instructions. For *Cristatella mucedo*, *Lophopodella carteri*, *Pectinatella magnifica*, *Phoronis ovalis*, *Plumatella* sp. and *Stephanella hina*, RNA was extracted at The University of Alabama using the E.Z.N.A. Mollusc RNA kit (Omega Bio-Tek, Norcross GA, USA) following the manufacturer's instructions. For samples processed at The University of Vienna, RNA-seq library preparation and sequencing were performed by the Next Generation Sequencing Facility at Vienna BioCenter core Facilities (VBCF) member of the Vienna BioCenter (VBC). Briefly, dual-indexed sequencing libraries were constructed using the NEBNext UltraTM II Directional RNA Library Prep Kit (#E7760, New England Biolabs, Frankfurt am Main, Germany) according to the manufacturer's instructions. For samples processed at The University of Alabama, dual-indexed sequencing libraries were prepared in-house using the Takara SMART-Seq HT kit with 1 ng of total RNA as input. The samples were then multiplexed and sequenced on an Illumina NovaSeq 6000 using an S4 flowcell with 2 × 150 bp paired-end reads with the S2 protocol at the VBCF or Psomagen (Cambridge, MA, USA).

Raw Illumina reads were first trimmed of adapters and low-quality sequences using Trimmomatic v. 0.39 [34] with default parameters. Read quality was checked before and after read trimming using FastQC v. 0.11.8 (www.bioinformatics.babraham.ac.uk/projects/fastqc/; last accessed 29 July 2021). The retained filtered and trimmed reads were assembled *de novo* using Trinity v. 2.8.4 [35], under default parameters, with the exception of a minimum transcript length of 200 nucleotides. The assembled transcripts of each sample were then translated with Transdecoder v. 5.02 (https://github.com/TransDecoder/TransDecoder/; last accessed 29 July 2021) with the –single_best_only option to predict and select the best single open reading frame (ORF) per transcript. Only transcripts with predicted ORFs of at least 100 amino acids (AAs) in length were kept for further analysis. To reduce redundancy in the predicted peptides, CD-HIT v. 4.8.1 [36] was applied using a threshold of 95% global similarity. The final transcriptome completeness of each sample was assessed using BUSCO v. 4.1.4 [37] against the conserved single-copy metazoan genes database (*n* = 978).

### (b) Orthologue assignment

We assembled and analysed two different matrices—one with *Fredericella sultana* 6 + 7 and one without these taxa, which was treated as the main matrix. Putatively orthologous groups shared among taxa were identified using OrthoFinder v. 2.5.2 [38] with an inflation parameter of 2.1. Groups produced by OrthoFinder (Orthogroup_Sequences directory) were processed through a modified version of the pipeline employed by Kocot *et al*. [39] in which sequences that were identical to longer sequences where they overlapped were removed from each orthogroup using UniqHaplo (http://raven.iab.alaska.edu/~ntakebay/; last accessed 29 July 2021), keeping the longest non-redundant sequence. To reduce missing data in the final matrix, only groups sampled for at least 31 of the 42 taxa (i.e. at least 70% of the taxa) were retained and sequences within them were aligned using MAFFT 7.310 [40] with the following options: –auto, –localpair and –maxiterate 1000. Mistranslated regions were cleaned using HmmCleaner [41] with the –specificity option. Then, alignments were trimmed using BMGE v. 1.12.2 [42] in order to remove ambiguously aligned and ‘noisy’ regions. AlignmentCompare (https://github.com/kmkocot/basal_metazoan_phylogenomics_scripts_01-2015/; last accessed 29 July 2021) was used to delete sequences that did not overlap with all other sequences by at least 20 AAs (starting with the shortest sequence meeting this criterion). Finally, genes sampled for a minimum of 31 taxa after these steps were retained.

In cases where two or more sequences were present for any taxon in a single-gene alignment, PhyloPyPruner 0.9.5 (https://pypi.org/project/phylopypruner/; last accessed 29 July 2021) was used to reduce the alignment to a set of strict orthologs. This tool uses a tree-based approach to screen each single-gene alignment for evidence of paralogy or contamination. An approximately maximum-likelihood tree was constructed for each alignment using FastTree 2 [43] with the -slow and -gamma settings. These alignments and trees were then used in PhyloPyPruner with the following settings: –min-support 0.9 –mask pdist –trim-lb 3 –trim-divergent 0.75 –min-pdist 0.01 –prune LS. Only orthogroups sampled for at least 76% of the total number of taxa were retained for concatenation. Paralogy frequency scores produced by PhyloPyPruner were evaluated to screen for exogenous contamination. Finally, we used BaCoCa v. 1.105 [44] to investigate the influence of shared missing data on phylogenetic reconstruction based on the degree of overlap in missing data between taxa. Determining the percentage of shared missing data can help to identify incorrectly reconstructed phylogenetic relationships.

### (c) Phylogenetic analyses

Phylogenetic trees were constructed using maximum likelihood (ML) and Bayesian inference (BI) on the main matrix produced by PhyloPyPruner. ML analysis was conducted on the partitioned data matrix using IQ-TREE2 v. 2.1.2 [45] with the best-fitting model of amino acid evolution for each partition (-m MFP). Topological support was assessed with 1000 ultrafast bootstraps. We also conducted ML analysis in IQ-TREE using the posterior mean site frequency (PMSF) model [46], with the LG + C60 + G + F model being specified. This method requires a starting tree to infer the site frequency model, thus, we used the previously generated IQ-TREE as a guide tree for PMSF analysis. Nodal support was assessed with 1000 replicates of ultrafast bootstraps. BI analysis was undertaken on an unpartitioned data matrix using PhyloBayes MPI 1.8 [47] with the site-heterogeneous CAT-GTR-G4 model. Two parallel chains were run for approximately 6000 cycles each with the first 1000 trees discarded as burn-in. A 50% majority rule consensus tree was computed from the remaining trees from each chain. Convergence of the PhyloBayes chains was determined based on the bpcomp maxdiff score which was 0, indicating that all chains had converged and reached stationary distribution. In PhyloBayes, a maxdiff score < 0.3 indicates that chains have converged and they have a good qualitative picture of the posterior consensus.

### (d) Ancestral state reconstructions (ASR)

Ancestral state reconstructions for colony morphology and statoblast types were performed in Mesquite 3.70 [48] using the ‘Trace Character History’ and ‘Likelihood Ancestral States' options with Mk1 model (Markov k-state 1 parameter model). The best ML tree inferred from the partitioned data matrix using IQ-TREE2 v. 2.1.2 was used as input tree for the ASR analyses. A morphological matrix for all analysed phylactolaemate species including two characters (colony morphology and statoblast types) was compiled. The colony morphology was assigned to two states (branching and clustered) and the statoblast types were assigned to three states (floatoblasts, piptoblasts and floatoblasts + sessoblasts).

### (e) DNA barcoding and pairwise distances

Mitochondrial cytochrome *c* oxidase subunit I (COI) was identified from the transcriptome assemblies of *F. sultana, Plumatella casmiana* and *P. repens* using blastn [49] and annotated using MITOS2 web server [50]. Genetic distances, uncorrected pairwise distances among the specimens of each of these three species were calculated in PAUP 4.0b10 [51] and ML-corrected pairwise distances were calculated in MEGA v. 11.O.11 using the maximum composite likelihood parameter with a gamma parameter of 1.0 [52].

### (f) Divergence time estimation

Divergence times were estimated in MCMCTree and codeml (both part of the PAML software package, v. 4.9) [53] using the unpartitioned data matrix. The best ML tree inferred from the partitioned data matrix using IQ-TREE2 v. 2.1.2 was used as a fixed input tree. A conservative age constraint was applied on the root of the input tree, with a soft minimum age of 529 Ma (millions of years), based on the oldest brachiopod fossil which is known from Cambrian Stage 2 [54,55] and a hard maximum age of 636.1 Ma, based on the origin of Spiralia/Lophotrochozoa (*sensu* [56–58]). The input tree was calibrated using age estimates of seven carefully selected fossils (for details, see electronic supplementary material, section 1 and table S2). To investigate the impact of calibration prior choice, we ran three MCMCTree analyses with different calibration strategies: (1) the truncated-Cauchy distribution ‘L’ with a soft minimum age and diffuse tail; (2) the skew normal ‘SN’ distribution with 97.5% cumulative probability of the distribution at the maximum age; (3) the uniform distribution ‘B’ in which fossil calibrations were constrained between the corresponding fossil age (minimum bound) and a maximum age equal to the maximum root age. The R package MCMCtreeR [59] was used to generate time priors for these three strategies. The independent rates model (clock = 2) was used to estimate divergence, for all three sets of analyses (for details of the substitution model and Markov chain Monte Carlo (MCMC) setting, see electronic supplementary material, section 2).

## 3. Results

### (a) Phylogenetic analyses

We sequenced the transcriptomes of 31 bryozoans and one phoronid and combined this dataset with publicly available transcriptomes of seven bryozoan species, one phoronid and two brachiopods. For the newly sequenced transcriptomes, BUSCO assessment with the Metazoa Odb10 database indicated that the completeness of the transcriptomes was higher than 81% for all species except for *Patinella* sp. and *Plumatella* sp. in which the BUSCO completeness scores were 20.2% and 53.9%, respectively (electronic supplementary material, figure S1). Our main data matrix included 1441 single-copy orthologous protein-coding genes (OGs) totalling 308 840 AAs with 24.10% missing data.

**Figure 1.**
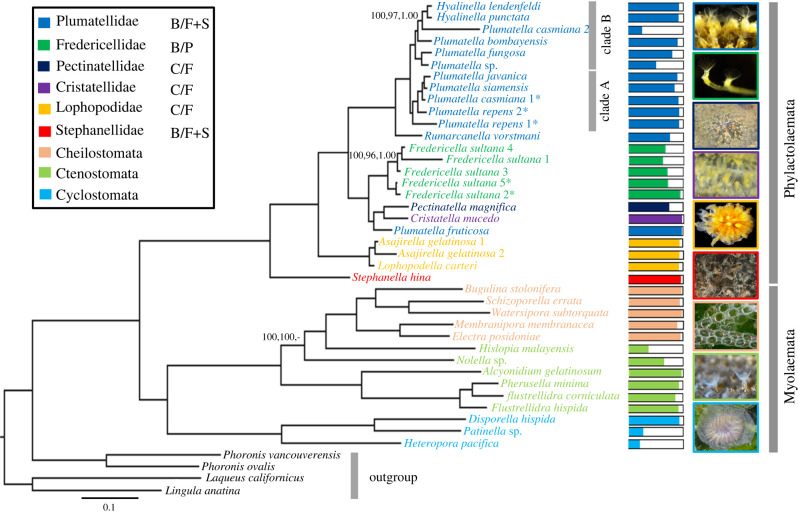
Maximum-likelihood phylogeny of Phylactolaemata based on the main data matrix using the partitioned analysis. Values on some nodes represent ML bootstrap support of the partitioned analysis, ML bootstrap support of the unpartitioned analysis with PMSF model and Bayesian posterior probabilities, respectively. Bootstrap support and Bayesian posterior probabilities are only shown for nodes that are not maximally supported by all analyses. Dashes in support values indicate that a relationship was not recovered. The asterisks with species names indicate that species were collected from Austria. Coloured bars show the proportion of genes sampled for each taxon. The scale bar represents 1 substitutional change per 100 AAs. Abbreviations next to phylactolaemate families represent charters mapping for colonies (B, branching; C, clustered) and statoblasts (F, floatoblast; S, sessoblast; P, piptoblast). (Online version in colour.)

Identical tree topologies for Phylactolaemata were inferred from all analyses based on the main data matrix with most of the nodes receiving maximal support (BI posterior probability, PP = 1.00 and ML bootstrap support, BS = 100) (ML based on partitioned data matrix with the best-fitting model for each partition, figure 1; ML based on unpartitioned data matrix with PMSF model, electronic supplementary material, figure S2; BI, electronic supplementary material, figure S3). Our results maximally support the sister group relationships between Phylactolaemata and Myolaemata. Within the latter clade, all analyses recovered Gymnolaemata as the sister group to Stenolaemata with maximal support. Similarly, all analyses recovered paraphyletic Ctenostomata.

Within Phylactolaemata, there is a clear early division between Stephanellidae on the one hand and a clade comprising all other phylactolaemates on the other. Lophopodidae is resolved as a monophyletic family and it is recovered as the sister group to a clade including Cristatellidae, Fredericellidae, Pectinatellidae and Plumatellidae. We recovered a clade comprising Cristatellidae, Fredericellidae, Pectinatellidae and *Plumatella fruticosa* from Plumatellidae. This clade has a sister group relationship to a clade comprising the remaining plumatellid taxa and it is split into two further lineages. Within the first lineage, there is a sister taxon relationship between *P. fruticosa* and a clade comprising Cristatellidae and Pectinatellidae. The second lineage consists of Fredericellidae and it is divided into two further groups. The first one comprising *Fredericella sultana* 2 & 5 from Austria while the second group includes *F. sultana* 1, 3 & 4 from Thailand. We also assembled and analysed the matrix including all publicly available transcriptomes of *F. sultana* from Austria in a separate analysis (electronic supplementary material, figure S4) and again all of which clustered together in one clade with maximal support. The comparisons of the uncorrected pairwise distances and the ML-corrected distances observed from the COI gene showed that divergence was high between the two *F. sultana* clades but was low within the sequences from the same clade (electronic supplementary material, tables S3 and table S4).

Plumatellidae is recovered as monophyletic with the exception of *P. fruticosa* clustering outside the rest of the plumatellids indicating that it is not a plumatellid. Within plumatellids, *Rumarcanella vorstmani* is recovered as the sister taxon of a clade comprising two groups (labelled as clade A and clade B in figure 1). Clade A includes *P. casmiana* 1 from Austria, *P. javanica*, *P. repens* 1 & 2 from Austria and *P. siamensis*. Within this clade, *P. javanica* and *P. siamensis* have a sister taxon relationship. Clade B comprises species from the genus *Hyalinella (H. lendenfeldi* and *H. punctata)* as well other species of genus *Plumatella* (*P. bombayensis*, *P. casmiana* 2 from Thailand, *P. fungosa* and *Plumatella* sp*.)*. Within this clade, *Hyalinella* is recovered as a monophyletic genus and *Plumatella* sp*.* is recovered as the sister taxon of *P. fungosa*. Our analyses showed that the two specimens of *P. repens* (from two Austrian localities) cluster separately from each other in clade A. The uncorrected pairwise genetic distance of the COI gene between *P. repens* specimens was (0.18) and the ML-corrected distance was 0.22. Similarly, *P. casmiana* specimens show a distinct placement with *P. casmiana* 1 (from Austria) falling in clade A while *P. casmiana* 2 (from Thailand) clustering in clade B. The uncorrected pairwise genetic distance of the COI gene between the *P. casmiana* specimens was (0.16) and the ML-corrected distance was 0.19. The results of the percentage of shared missing data analysis showed that shared missing data between taxon pairs are randomly distributed among the taxa and nothing stand with respect to *P. casmiana or P. repens* specimens (electronic supplementary material, tables S5 and S6), although there are differences in data presence/absence for the sampled specimens, consistent with the observed differences in branch lengths for these taxa*.* However, *P. repens* 2 has a high paralogy frequency value, which could indicate exogenous contamination, and thus we re-ran the ML analysis excluding *P. repens* 2 (electronic supplementary material, figure S5). Removing this taxon did not impact the overall topology.

In the light of the reconstructed phylogeny, we inferred the evolution of key phylactolaemate characters including colony morphology and statoblast types. Given the reconstructed phylogeny, it is clear that branching colony forms represent the plesiomorphic condition, with clustered or gelatinous forms having evolved in Lophopodidae and within the *Pectinatella*–*Cristatella*–*P. fruticosa* (PCP) clade. Our ASR analyses also show that branching colony type is ancestral state within the Phylactolaemata (electronic supplementary material, figure S6). In terms of statoblast types, floatoblasts are inferred to be the ancestral type whereas, sessoblasts seem to have evolved twice independently in stephanellids and the last common ancestor of the PCP and plumatellid clades. Alternatively, sessoblasts could also have evolved only once and have been lost in lophopodids. However, the ancestral states of statoblast types remain unclear in our ASR analyses (electronic supplementary material, figure S7).

### (b) Divergence time analysis

**Figure 2.**
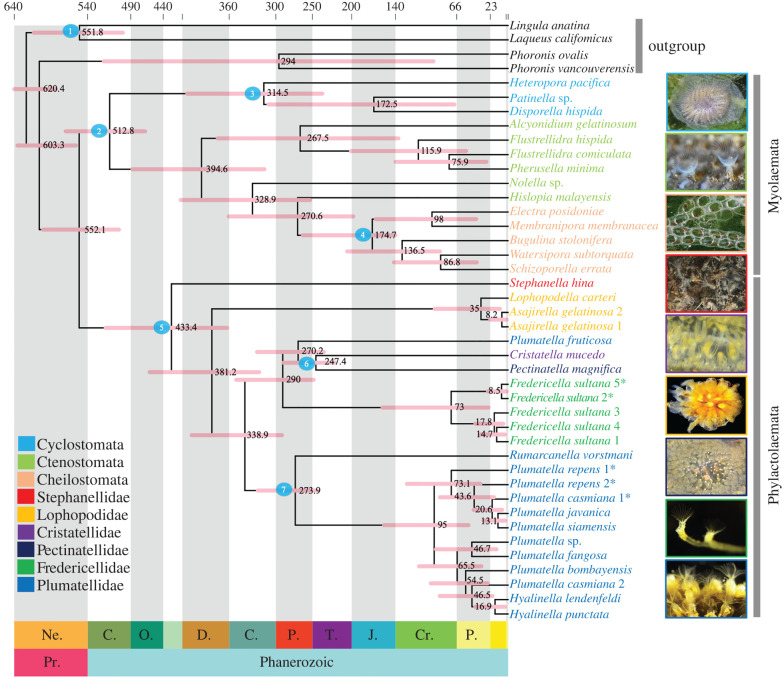
Time-calibrated phylogeny of Phylactolaemata using MCMCTree based on the main data matrix. Posterior distributions, based on the truncated-Cauchy priors. A time scale in Ma is shown above the tree, with geographical periods labelled below the tree. Node bars represent 95% confidence intervals of age estimates and raw numbers for mean. Numbered circles represent nodes with fossil calibrations correspond to the numbers in electronic supplementary material, table S2. (Online version in colour.)

All three divergence time analyses resulted in largely congruent age estimates (truncated-Cauchy, figure 2; skew normal, electronic supplementary material, figure S8; uniform, electronic supplementary material, figure S9; comparison of age estimates for key nodes from the three different calibration strategies, electronic supplementary material, figure S10). Hence, further reporting of results and discussion are based on the truncated-Cauchy analysis. Our divergence time estimates show that bryozoans diverged from their most recent common ancestor in the Ediacaran at approximately 603.3 Ma (95% credibility interval [CI], 556 to 631.2 Ma). The major bryozoan clades (Myolaemata and Phylactolaemata) began to split at∼552.1 Ma (CI, 500.6–598.1 Ma). Within Myolaemata, the first divergence, namely, the separation of Gymnolaemata from Stenolaemata, is estimated to have occurred in the late Cambrian (approx. 512.8 Ma; CI, 476–569.1 Ma), followed by divergence of Gymnolaemata in the Middle Devonian (approx. 394.6; CI, 313.6–483.7 Ma). The diversification of Cyclostomata and Cheilostomata took place in the Carboniferous at the Middle Pennsylvanian (approx. 314.5; CI, 239.2–413 Ma) and Early Jurassic (approx. 174.3 Ma; CI, 145–264.1 Ma), respectively.

Within Phylactolaemata, the split between Stephanellidae and the remaining phylactolaemate taxa occurred in the Silurian at Llandovery epoch (approx. 433.4 Ma; CI, 361.7–517.6 Ma). The second divergence in the phylactolaemate tree between Lophopodidae and the remaining taxa occurred during the Late Devonian (approx. 381.2 Ma; CI, 320.4–461.2 Ma). Among the remaining phylactolaemate families, Fredericellidae evolved from its most recent common ancestor in the Permian at the Cisuralian epoch (approx. 289.9 Ma; CI, 250.2–349.8 Ma). The clade that unites Cristatellidae and Pectinatellidae diverged from *P. fruticosa* during the Permian at the Guadalupian epoch (approx. 270.1 Ma; CI, 237.1–323 Ma), and the divergence between *Cristatella mucedo* and *Pectinatella magnifica* occurred in the Middle Triassic (approx. 247.4 Ma; CI, 221.9–289.1 Ma). Plumatellidae evolved from its most recent common ancestor in the Carboniferous at the Middle Mississippian epoch (approx. 338.9 Ma; CI, 300–407.1 Ma) and began to diverge in the Middle Permian (approx. 274 Ma; CI, 246.3–322 Ma). The first diversification of most extant phylactolaemate families mainly occurred in the Palaeogene and Neogene periods.

## 4. Discussion

### (a) Phylogenetic relationships of Phylactolaemata

Here, we present a phylogenetic framework for Bryozoa with special emphasis on Phylactolaemata using phylogenomic analyses based on transcriptomic data in which the interrelationships among the phylactolaemate families are fully resolved and strongly supported across all analyses. The relationships between the main bryozoan clades are also well resolved with Phylactolaemata recovered as the sister group to Myolaemata, consistent with previous molecular studies based on fewer loci [7,8]. Likewise, our results confirmed the sister group relationships between Gymnolaemata and Stenolaemata and recovered a paraphyletic Ctenostomata as previously suggested [7,8].

Our phylogenomic analyses recovered Stephanellidae as the sister group to all other phylactolaemates and confirmed that Lophopodidae represents the second offshoot of the phylactolaemate tree. Previous morphology-based phylogenetic studies of Phylactolaemata suggested that *Stephanella* represents a plesiomorphic taxon as this genus has retained putatively ancestral features particularly the cruciform budding and colony growth pattern, and a relatively small number of tentacles [14,20]. However, a precise phylogenetic position of *Stephanella* has not been indicated as this genus has a strange combination of plesiomorphic and apomorphic characters [14]. A recent morphological study by Schwaha & Hirose [60] suggested that there are several possible apomorphic details characteristic of *Stephanella hina* supporting the distinct placement of this species in a separate phylactolaemate family. The relationship of Lophopodidae to other phylactolaemates was also unclear. The Lophopodidae was traditionally regarded as a derived group from Plumatellidae as either closely related to *Gelatinella* based on the mammillation on the surface of *Lophopus* and *Gelatinella* statoblasts [20] or closely related to *Hyalinella* based on the synapomorphies of the absence of sessoblasts and the nonbuoyant floatoblasts [14]. Our results strongly reject the placement of Lophopodidae as a derived group and instead support the earlier branching position of Lophopodidae. By contrast to morphological studies, molecular phylogenies based on few genes consistently suggested that both Stephanellidae and Lophopodidae represent early branches in phylactolaemate phylogeny. However, the exact phylogenetic position of these two families remained uncertain with Stephanellidae and Lophopodidae either recovered as sister groups [8,23,26] or Stephanellidae recovered as a sister group to all other taxa with Lophopodidae falling out as the second offshoot of the phylactolaemate tree in the maximum-parsimony analysis of Hirose *et al*. [23], though without significant support. Our results show clearly that Stephanellidae is the sister taxon of the remaining phylactolaemate lineages and Lophopodidae falling as the second offshoot of the phylactolaemate tree in support of the results by Hirose *et al*. [23].

Of particular interest, all of our analyses firmly place *P. fruticosa* outside Plumatellidae. Although this finding was reached in a previous molecular study [25], the position of *P. fruticosa* was unresolved. Our results, for the first time, recovered *P. fruticosa* as the sister taxon to a well-supported clade comprising Cristatellidae + Pectinatellidae (PCP-clade). Morphologically, *P. fruticosa* shows a mosaic of plumatellid and fredericellid characters [3], but distinct similarities to Cristatellidae or to Pectinatellidae currently remain unknown and require further study. Particularly supportive of a closer relationship to *Fredericella* is the erect branching colony morphology of *P. fruticosa* and similarities in larval morphology [3]*.* Our phylogenies are in favour of this notion with *Fredericella* being the sister clade to the PCP grouping. It can be concluded that a branching type of colony morphology was common to the ancestor of Plumatellidae and the Fredericellidae-PCP clade and that the ‘gelatinous’-clustered type of Cristatellidae and Pectinatellidae appeared later during phylactolaemate evolution.

The relationship of Fredericellidae to other phylactolaemate families has always been considered problematic [7,8,22,26]. In traditional morphological studies, Fredericellidae was regarded as the earliest branch among extant phylactolaemates since it shows very simple statoblasts (piptoblasts), chitinized colonies, a dendritic colony branching pattern, a relatively small number of tentacles arranged in a circle (as in myolaemates), and relatively small yolk granules [14,20,21]. On the other hand, fredericellids were shown to be relatively late-branching taxa based on molecular studies, though their relationship to other phylactolaemates was not precisely resolved [8,22,23,26]. Notably, we recovered a novel phylogenetic placement for Fredericellidae as the sister group to a clade comprising *P. fruticosa* Cristatellidae + Pectinatellidae with strong support. Fredericellidae is represented by five *Fredericella* specimens (*F sultana* 1, 3 & 4 from Thailand and *F sultana* 2 & 5 from Austria), which are divided into two clades according to their geographical distribution. This genetic structure is also supported by the pairwise sequence comparisons of the COI gene since the genetic differences between the two *F. sultana* clades were larger than the pairwise differences of sequences from the same clade indicating that *F. sultana* in our analyses might belong to different species. Obvious geographical patterns in the phylogenetic topologies within *F. sultana* have also been shown by Hartikainen *et al*. [25] with *F. sultana* being divided into four well-supported lineages. Unfortunately, we were not able to include any specimens from the second fredericellid genus, *Internectella*, which is also the only true fredericellid with a floatoblast. Future inclusion of this genus might further resolve the relationship of the fredericellid and PCP clade.

Our results show that *P. fruticosa* falls outside the rest of plumatellids and thus is not a plumatellid. Likewise, our analyses firmly place *Hyalinella* within *Plumatella,* rendering the latter genus paraphyletic, consistent with previous molecular phylogenetic studies [22,23,25]. Within the main plumatellid group, *R. vorstmani* is recovered as the sister taxon to the remaining plumatellid taxa with strong support, consistent with the molecular phylogenies of Hirose *et al*. [23] and Waeschenbach *et al*. [8]. Both studies showed that *Rumarcanella vorstmani* *+* *R. minuta* represent an early branch within Plumatellidae and that the genus *Rumarcanella* is most likely to be sister-taxon to the remaining plumatellids. Apart from *R. vorstmani*, the remaining plumatellids are clearly divided into two well-supported clades (clade A and clade B, figure 1) in which the species relationships received high resolution. This phylogenetic pattern does not precisely match any previous studies [8,25,61]. Importantly, our results show that cryptic speciation probably occurs in two *Plumatella* species (*P. repens* and *P. casmiana*). *Plumatella casmiana* is represented by single isolates from Austria (1) and Thailand (2) whose statoblasts are indistinguishable, but the isolates of each species are deeply distinct from each other with uncorrected pairwise distances of 0.16 and ML-corrected distance of 0.19 for the COI gene. Colonies of *P*. *casmiana* normally appear as flat, rounded patches of densely arranged zooids oriented towards the periphery. The ectocyst is flexible and variably encrusted. On limited substrata, zooids may become erect and fused together while a basal layer of the colony becomes sclerotized and stiff [62]. In Thailand, a second colony form has been reported [63]. Zooids form a ragged and loosely organized assemblage. The ectocyst is stiff, sclerotized, amber in colour, and unencrusted near the distal ends of the zooids, this condition spreading to all parts of the colony as it ages. On limited substrata the zooids become crowded but are never fused. In both instances, the lophophores bear fewer than 26 tentacles, and the statoblasts appear morphologically indistinguishable. *P*. *repens* specimens from two Austrian localities also do not cluster together with uncorrected pairwise distances of 0.18 and ML-corrected distances of 0.22 for the COI gene, which indicates that there are two different species, possibly an undocumented and not properly recognized plumatellid similar to *P. repens*, such as *P. rugosa* [15]. This finding contradicts the suggestion of Hartikainen *et al*. [25] that genetic divergence within *Plumatella* is usually accompanied by morphological differences. Finally, within clade A, *P. javanica* and *P. siamensis* cluster together as sister taxa with strong support. Phylogenetic relationships of these two species have not been investigated in previous morphological and molecular phylogenetic studies.

### (b) Divergence time analysis (discussion)

In this study, we attempted to estimate times for the origin and diversification of bryozoans with special emphasis on Phylactolaemata based on phylogenomic analyses. Our results indicate that bryozoans evolved from their most recent common ancestor as early as the Ediacaran (approx. 603.3 Ma) and diverged into Myolaemata and Phylactolaemata at approximately 552.1 Ma in the end of the Ediacaran. However, the earliest occurrence of bryozoan fossils (*Protomelission gatehousei* from Australia and South China) [4] and (the Age 4 Cambrian Harkless bryomorph fossil from western United States) [5] are dated to the early Cambrian, which is younger than the age observed from our molecular clock analysis and also falls outside the range of the 95% CI (556.0–631.2 Ma) for the origin of bryozoans in our analysis. Thus, bryozoans may have evolved well before their first appearance in the fossil record at the early Cambrian. Our divergence time estimate for the origin of bryozoans is younger than that of the time-calibrated phylogeny of Metazoa by Erwin [64] where the bryozoan lineage stretches back into the early part of the Ediacaran (approx. 630.0 Ma). This age falls within the range of the 95% CI (556.0–631.2 Ma) for the origin of bryozoans in our analysis. However, a recent study by Orr *et al*. [9] suggested that bryozoans diverged from their most recent common ancestor as early as the Ediacaran, consistent with the results of our study. Divergence time estimates show that phylactolaemates have diverged from their most recent common ancestor in the end of Ediacaran with credibility interval extended to the late Cambrian (500.6–598.1 Ma). A comparable age estimate is also shown by Orr *et al*. [9] in their sensitivity analyses (using different clock models and calibrations). The diversification of Myolaemata into Stenolaemata and Gymnolaemata occurred at approximately 512.8 Ma (CI, 476–569.1 Ma). This late Cambrian diversification is supported by the presence of six major bryozoan orders belonging to Stenolaemata and Gymnolaemata during the Lower Ordovician period with considerable diversity [65–67]. The divergence times of the main myolaemate groups (Cheilostomata, Ctenostomata and Cyclostomata) inferred in this study are largely in agreement with those observed by Orr *et al*. [9].

The first divergence within the phylactolaemates occurred around 433.4 Ma in the Silurian, which is substantially older than the earliest phylactolaemate fossil evidence of statoblasts from the Upper Permian of Russia [17]. The age of the first phylactolaemate fossil also falls outside the range of the 95% CI (361.7–517.6 Ma) of this node. However, the fossilization of phylactolaemates has been hampered by the total lack of a calcified skeleton in this group. Likewise, the absence of earlier fossil evidence might be attributed to the assumption that statoblasts represent an adaptation of living in freshwater environments, whereas the earliest phylactolaemate ancestors were probably marine and thus did not produce statoblasts [68]. Previous molecular sequence analyses recover a sister group relationship between Phylactolaemata and Myolaemata [7–9]. Therefore, it has been suggested that phylactolaemate fossils should occur alongside those of myolaemates [68]. The results of our analyses are consistent with this suggestion since phylactolaemates are inferred to have evolved from their most recent ancestor as early as the Ediacaran and began to diverge in the Early Silurian, though with a wide credibility interval.

## 5. Conclusion

By employing a phylogenomic approach with dense taxonomic sampling, we were not only able to provide a fully resolved phylogeny for extant Phylactolaemata but also determine the origin and initial diversification of extant phylactolaemates. The overall phylogenetic hypothesis observed from our analyses support previous molecular phylogenies in that Stephanellidae and Lophopodidae are early branching phylactolaemate families and Fredericellidae and Plumatellidae are more derived groups. Furthermore, our results give clarity to the ambiguous relationships among phylactolaemate families and provide high resolution for species relationships. Our results suggest that cryptic speciation probably occurs in *F. sultana* and in two species of *Plumatella* (*P. repens* and *P. casmiana*). Further studies with more taxon sampling of these species are required to confirm this relationship. Our divergence time estimates showed that bryozoans have a Precambrian origin coinciding with the recent finding that bryozoan fossils go back to the early Cambrian and supporting the recently published dated phylogeny by Orr *et al*. [9]. The first diversification within Phylactolaemata occurred in the Silurian and the radiation of most phylactolaemate families have mainly occurred in the Palaeogene and Neogene periods highlighting post-extinction diversification. The lack of fossil evidence from Cristatellidae, Fredericellidae and Lophopodidae as well as the absence of molecular clock data on Phylactolaemata prior to the present study represented a challenge for our study by limiting the available calibration points, resulting in wide credibility intervals for some nodes. Additional new phylactolaemate fossils have a high potential for defining new calibration points for future studies.

## Data Availability

Data available from the Dryad Digital Repository and from Figshare [69,70].
